# Radiology and multi-scale data integration for precision oncology

**DOI:** 10.1038/s41698-024-00656-0

**Published:** 2024-07-26

**Authors:** Hania Paverd, Konstantinos Zormpas-Petridis, Hannah Clayton, Sarah Burge, Mireia Crispin-Ortuzar

**Affiliations:** 1 Cambridge University Hospitals NHS Foundation Trust, Cambridge, UK; 2 Department of Oncology, University of Cambridge, Cambridge, UK; 3 Cancer Research UK Cambridge Centre, University of Cambridge, Cambridge, UK; 4 Fondazione Policlinico Universitario Agostino Gemelli IRCCS, Rome, Italy

**Keywords:** Cancer imaging, Predictive markers

## Abstract

In this Perspective paper we explore the potential of integrating radiological imaging with other data types, a critical yet underdeveloped area in comparison to the fusion of other multi-omic data. Radiological images provide a comprehensive, three-dimensional view of cancer, capturing features that would be missed by biopsies or other data modalities. This paper explores the complexities and challenges of incorporating medical imaging into data integration models, in the context of precision oncology. We present the different categories of imaging-omics integration and discuss recent progress, highlighting the opportunities that arise from bringing together spatial data on different scales.

## Introduction

The advent of modern machine learning has completely shifted the landscape of predictive modelling for precision oncology. Applications within medical imaging in particular have boomed, achieving notable success. In parallel, there has been a growing interest in data integration, thanks to the development of datasets that span from the molecular scale of genes all the way to the macroscopic scale of medical scans. These datasets offer a holistic view of the tumour and its surrounding environment, with the potential of enabling tailor-made therapies for individual patients. However, applications that integrate radiological imaging are still comparatively underdeveloped. Instead, most of the attention has focused on the fusion of multi-omic molecular profiles, which already provide very rich, high-dimensional information. Multi-omic datasets also have the advantage of sharing the same subcellular physical scale. Sometimes, thanks to the availability of the data, studies have been extended to include digital pathology, bringing in a new scale—cells and their microenvironment—and introducing the need to perform two-dimensional spatial analysis^1–3^.

Including radiological images, however, is a completely different challenge. They capture the entirety of the disease, macroscopically and in three dimensions. If the cancer is metastatic, usually the majority of the lesions will be included in the scan, introducing a source of heterogeneity that is inaccessible for biopsies. The resolution of a typical computed tomography (CT) or magnetic resonance imaging (MRI) scan is of the order of a millimetre, orders of magnitude larger than the pixels in a microscopy image. Moreover, scanners and image acquisition protocols can result in noticeable and highly variable noise and image artefacts, threatening the generalisability of any result. These differences are challenging, but they are also the reason why including radiological imaging in fusion models can add significant value, as no other data type contains comparable information of such critical importance for the patient’s journey. In addition, advanced imaging methods can provide a wealth of additional data: from in-vivo functional imaging using positron emission tomography^4^, to measures of blood flow with MRI perfusion techniques^5^, physical properties of tissues, such as liver stiffness with MR elastography^6^, and molecular displacement with diffusion weighted imaging^7^. Due to its non-invasive nature, imaging is far more often performed at regular intervals in clinical practice compared to other data modalities (e.g. biopsies), bringing temporal dimension to data integration.

At a time of rapid development of artificial intelligence (AI) models, we want to shed light on the complexities of integrating medical imaging data in data integration models for precision oncology. The majority of the work in this space relies on well-established machine learning (ML) methods that can provide the robustness required. We illustrate this point in Section “Data fusion”, which reviews the literature on the prediction of clinical endpoints using imaging-omics fusion. We also discuss current approaches and challenges in data translation (Section “Data translation”) and data aggregation (Section “Data aggregation”), which are parallel but equally critical elements of the data integration paradigm for precision oncology.

## Types of data integration

**Fig. 1:**
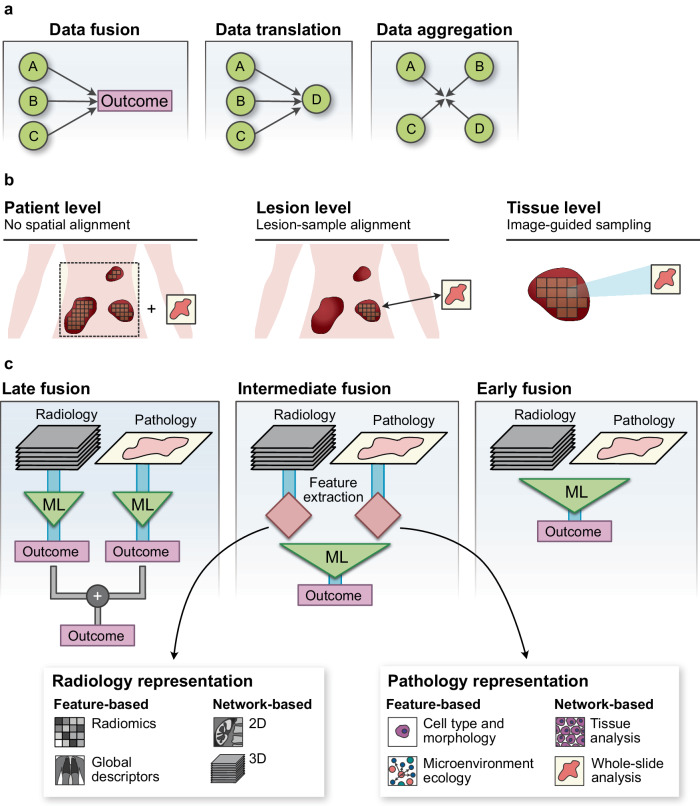
Types of data integration. **a** Simplified illustration of the three types of data integration processes considered in this review, classified as a function of the task and its objective. Data sources of different modalities are depicted in the figure with the letters A-D. Clinically-relevant endpoints are indicated by the label 'Outcome'. **b** Representative examples of the different levels of data fusion based on the physical scale at which the data is aligned, including patient-level (data sources are treated as independent), lesion-level (imaging features are matched to corresponding samples), or tissue-level (tissue samples are taken from specific locations using image guidance) fusion. **c** Different types of data fusion architectures in terms of the stage at which data streams are combined. The figure uses radiology and pathology images as an illustration. Clinically-relevant endpoints are indicated by the label 'Outcome'.

Data integration is a broad term that can refer to different aspects or objectives within the process of bringing different data types together. In this review we explore three broad types of data integration; namely data fusion, data aggregation, and data translation, as illustrated in Fig. 1a.

We use the term *data fusion* to refer to the combined use of data from diverse sources in a single model, with the aim to predict clinical endpoints such as survival or treatment response with higher accuracy than would be possible using each source separately. For example, a data fusion model may combine MRI scans, genomics and digital pathology to predict progression-free survival. The main challenges for data fusion are related to the disparity of the data modalities, which makes modelling difficult; and the lack of large enough datasets that contain all the data sources of interest. Data fusion is the main focus of this review, and we will explore it in detail in Section “Data fusion”.

The term *data translation* is closely related to data fusion, and refers to models that predict the information from a given data type by using all the others jointly in a predictive model. For example, a data translation model may use clinical data and radiomics to predict histopathologic subtype. Data translation is particularly useful as an explainability tool, or for creating surrogate markers of data types which may be difficult to obtain. We explore data translation briefly in Section “Data translation”.

Finally, *data aggregation* refers in this context to the systematic process of collecting, preparing, and presenting diverse datasets for subsequent analysis. For example, a data aggregation framework may link electronic health records with digitised histopathology slides, to enable the deployment of computational pathology models. Although data aggregation is a necessary step prior to other data integration tasks (i.e. data fusion or data translation), it can also be a goal in itself, for example enabling clinicians to access multiple data modalities seamlessly to improve direct patient care, or to create online databases and biobanks. The main challenges of data aggregation are typically related to systems and processes, and include data integrity, system interoperability, and anonymisation. We provide an overview of the goals and challenges of data aggregation in Section “Data aggregation”.

## Data fusion

In data fusion models, the information from multiple modalities is combined to obtain a better predictor of a given clinical endpoint, which could be tumour detection, diagnosis, treatment response, or outcome. They represent the essence of the personalised precision oncology paradigm—in other words, the idea that the treatment journey of a cancer patient can be tailored using their unique, individual, multimodal data profile.

One of key decisions for data fusion models is the level at which the different modalities will be integrated, namely at the patient, lesion, or tissue levels (Fig. 1b). Typically, each data modality is treated as providing an independent patient-level representation, with no attempt at spatial co-registration. When the data is sufficiently annotated, it is also possible to provide lesion-level alignment, by matching specific samples with the images corresponding to the lesion they were extracted from. Tissue-level alignment, in which samples can be traced to specific locations within the lesion, normally requires advanced spatial techniques or careful sampling using image guidance, as discussed in Section “Data translation”.

In addition, fusion models are typically divided into late, intermediate or early fusion^8,9^, as illustrated in Fig. 1c. In late fusion approaches, single-modality models are developed independently and merged in the final layers. This reduces the complexity of the training process and may also make it possible to re-utilise previously trained models. In early fusion, all modalities are processed simultaneously within a single deep learning architecture. Models where data fusion happens earlier typically require more data for learning but may be able to capture cross-modal interaction better^10–12^. More work is needed to develop and benchmark performance of such methods in the biomedical domain. Intermediate fusion models take early representations of the data for further learning. Fusion models that integrate medical images often opt for late or intermediate fusion approaches, in which imaging information is first collapsed down to a reduced, uni-dimensional set of features. One option is to generate lists of handcrafted features based on fixed definitions, which can be motivated by clinical or biological expectations (e.g. presence of disease in a certain area), or by mathematical characterisations of the texture of the image (e.g. Haralick features)^13^.

Alternatively, these representations can be based on pre-trained deep learning models that act as feature extractors, which is particularly useful if the primary dataset is limited in size, as is often the case in clinical studies. Pre-training can also be used as a general approach, whereby models are trained on an auxiliary task and then fine-tuned on the dataset of interest, in a process known as transfer learning^14^. Radiomics feature extraction through transfer learning has been successfully applied in the development of models for computer-aided diagnosis as well as outcome prediction in a variety of cancer types, including lung^15,16^, breast^15^, gastric^17^ and brain tumours^18^. Transfer learning can also be used for medical image segmentation, with the potential to reduce the need for manual annotation of the datasets and even to increase the segmentation accuracy, although consideration must be given to the pre-training and target dataset domains^19–21^. Self-supervised learning, a different type of pre-training that does not require annotated labels, has also been more recently shown to be effective^22,23^.

### Literature review

We conducted a PubMed search to identify predictive data fusion studies including medical imaging (18 June 2023). We focused our search on models predicting clinical outcome only, such as overall survival and progression-free survival^24^; and treatment response metrics, such as RECIST^25^. The exact query terms can be found in the Supplementary Table 1. Other related tasks such as diagnosis, which are closer to the data translation category, were not included in our search.

**Fig. 2:**
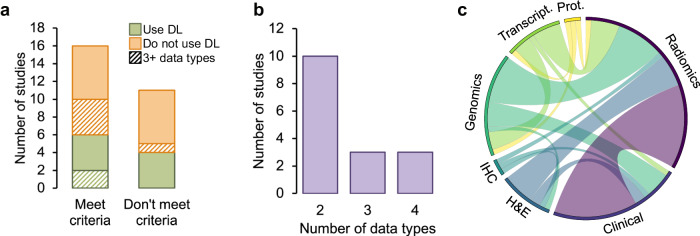
Review of data fusion. **a** Breakdown of the papers identified according to whether they satisfy the inclusion criteria; use deep learning or not; and integrate three or more data types or not. **b** Number of data types integrated in each of the studies that satisfied all the inclusion criteria. **c** Circos plot illustrating the co-occurrence of the different data types in the studies that satisfied all the inclusion criteria. Prot. = Proteomics, Transcript. = Transcriptomics.

Our search identified a total of 27 studies. Their details are summarised in the Supplementary Table 2. Upon manual review, we excluded a total of 11 studies (one review, one abstract book, one database report, and eight studies that did not include an outcome prediction model). Of the remaining 16 papers that met the inclusion criteria, the majority (10) only integrated two data modalities, as shown in Fig. 2. Three studies integrated three data modalities, and three integrated four data modalities. Of the 16 studies that met the criteria, only six used deep learning, of which only two integrated more than two data modalities. The limitations of our review are discussed in Section “Discussion and outlook”.

#### Machine learning vs deep learning

The majority of studies we reviewed use a late fusion approach, with separate models being trained for each data modality, followed by an integration layer of typically minimal complexity. Two studies conduct separate analyses (including ML models) for each single modality and then combine the derived signatures by statistical analysis^26,27^. For example, Wang et al.^26^ use four different data modalities (haematoxylin and eosin slides, radiomics, immunohistochemistry and clinical data) to predict survival in colon cancer, but they derive a separate risk score for each modality and then integrate them into a combined nomogram by multivariate analysis.

Two papers with a late fusion approach use traditional ML methods for the integration step. For example, Feng et al.^28^ derive separate radiomics and pathomics signatures to predict response to neoadjuvant chemotherapy in rectal cancer, and combine them into a predictive model using a support vector machine (SVM). Wang et al.^29^ also use SVM in a late fusion model combining a radiomics score (derived by a deep neural network) and clinical data to predict EGFR genotype in lung cancer. Five other studies use traditional ML methods for data integration with an intermediate fusion approach, including logistic regression^30^, random forest^31–33^, SVM^33,34^ and LASSO^33^.

Several studies take an indirect approach to data integration. Iwatate et al.^35^ use a two-step approach for survival prediction: they predict molecular status (p53 and PD-L1) of pancreatic ductal adenocarcinoma from radiomics using an XGBoost algorithm, and then show a difference in survival between the predicted groups by conventional statistical analysis. Unsupervised ML is utilised in some studies, e.g. Hoivik et al.^36^ perform unsupervised clustering of radiomics features extracted from MRI scans of endometrial cancer, and then show a statistical difference between the groups in several domains including survival, clinicopathological, transcriptomic and genomic features. Liang et al.^37^ also use an indirect approach: they derive two clusters of patients with renal cell carcinoma based on their transcriptomics data, show a statistical difference in survival between the two clusters, and then build a machine learning model from radiomics features to predict these two clusters.

Only three studies use deep learning approaches for data integration, among which two use representation learning. Zhang et al.^38^ use representation learning to extract deep radiomics features from MRI scans and integrate them with clinical data to predict microsatellite instability in rectal cancer. In contrast, Vanguri et al.^39^ use radiomics features as input to a multiple-instance learning model to predict immunotherapy response in non-small cell lung cancer, integrating the radiomics with genomics and immunohistochemistry data. Interestingly, Li et al.^40^ use an intermediate fusion approach, in which the algorithm characterises the multimodal interactions between histology and radiology features (both handcrafted and convolutional neural network (CNN) generated) by learning hierarchical co-attention mappings for the two modalities. This integrated embedding as well as the original radiomics data are then passed on together to the transformer layers for survival prediction in gastric cancer.

Deep learning was more commonly used as an isolated part of the image processing pipelines, including image segmentation and radiomics feature extraction. Image segmentation is potentially a major barrier to implementation of radiomics models in clinical practice. Manual delineation of all disease sites is necessary for radiomics feature extraction in traditional ML approaches, but it is time-consuming and requires radiologists’ expertise. Although the majority of studies in this systematic review include a manual segmentation step^26–28,31–35,38,39^, four papers successfully utilise deep learning models to automate the segmentation task^29^^,36,40^^,41^. Moreover, Hoivik et al.^36^ compare both approaches, concluding that an automated segmentation model reproduces the same results as manual segmentation. The growing success of deep learning based segmentation algorithms for both tumours and healthy organs is lowering the entry barrier for the inclusion of imaging in data integration studies, as well as making these multimodal models easier to implement in clinical practice.

A further issue when considering traditional ML versus deep learning approaches is the method of radiomics feature extraction. Only deep learning allows for autonomous extraction of features from raw radiological data - otherwise the model depends on a set of handcrafted features. Among papers using traditional ML for data integration, all but one^29^ use a handcrafted set of radiomics features, often extracted with open-source software such as pyradiomics. Only two papers use deep learning (CNN) for radiomics feature extraction^29,38^, and one utilises both handcrafted and deep learning (CNN) features^40^. Deep learning is also used for pathology feature extraction in five studies (CNN^26–28,39^, vision transformer^40^).

Using traditional ML versus deep learning also has implications on interpretability techniques which can be applied. Simple models such as logistic regression are usually very transparent and can provide a quantitative explanation of the model (e.g. feature coefficients). However, the implementation, or at least reporting, of such techniques is variable. In this review, most studies using traditional ML identify the list of radiomics features selected by the model, but further exploration and biological interpretation of the selected features are often limited. Some interpretability approaches include quantification of the importance of radiomics features for the model’s predictive power^31^, and analysis of a correlation heatmap between radiomics features and data from other modalities (including hallmark genes and tumour microenvironment)^34^. In contrast, deep learning approaches can be less transparent, however new interpretability approaches are being developed to shine light into the model’s processes. For example, of the three papers using deep learning for the data integration step, two^38,40^ include saliency or attention maps superimposed on the original radiological image to gain insight into the decision-making process. The development of such interpretability approaches for deep learning techniques is particularly important if these models, often more powerful than traditional approaches, are to be implemented in clinical practice.

#### Integration with computational pathology

Five studies in our literature review include integration with computational pathology, which is an evolving technique based on the analysis of digitised histopathology images, including haematoxylin and eosin (H&E) and immunohistochemistry (IHC) slides^42^. This number excludes studies in which pathology-based features are included as part of a list of clinicopathological variables^29,30,33,35,36,38^, instead of using the pathology slides as input to a computational method.

Boehm et al.^27^ use a weakly-supervised ResNet-18 CNN for tissue classification and feature extraction from whole slide images (WSIs). These histopathological features are then integrated with radiomic, genomic and clinical data by a Cox model in a late fusion approach.

Late fusion is also used by Wang et al.^26^, who develop a pathomics signature from WSIs using a weakly-supervised patch-level CNN. This pathomics signature is then integrated by multivariate analysis with three other single-modality signatures (radiomics, IHC and clinical data) to develop a combined nomogram patient survival prediction in colorectal cancer.

Feng et al.^28^ use two WSI analysis methods: an open-source software (CellProfiler) to extract nucleus features, and VGG-19 to extract microenvironment features. They develop two pathomics signatures (nucleus and microenvironment, respectively), and combine them with the radiomics signature by SVM in another late fusion approach to predict treatment response to neoadjuvant chemoradiotherapy in locally advanced rectal cancer.

Vanguri et al.^39^ also use deep learning for tissue classification by means of a DenseNet classifier in combination with the commercially available HALO AI software for feature extraction. These histopathological features are then integrated with radiomics and genomics in an attention-based deep multiple-instance learning model to predict treatment response to immunotherapy in non-small cell lung cancer.

Li et al.^40^ use a hierarchical vision transformer to extract the patch- and region-level features in WSIs. As described above, they then apply an intermediate fusion layer which learns hierarchical co-attention mapping between histology and radiology features and subsequently use multiple-instance learning to predict survival in gastric cancer.

#### Integration with genomics

Most studies that include genomics in their data fusion models do so by focusing on established biomarkers with known predictive power.

Targeted single-gene sequencing is commonly included, e.g. EGFR in Vanguri et al.’s^39^ non-small cell lung cancer study or VHL in Zeng et al.’s^32^ renal cell carcinoma study. Yi et al.^33^ focus on only one gene (SULF1) and investigate its 12 single-nucleotide polymorphisms (SNPs); the SNPs are then included as genomic features in a model predicting platinum resistance in ovarian cancer.

A few studies take a different approach. Boehm et al.^27^ determine the homologous recombination deficiency (HRD) status of patients by investigating COSMIC signatures as direct evidence of HRD, in addition to sequencing for known predisposing variants (e.g. in BRCA1 and BRCA2 genes). The HRD status is then combined with computational pathology and radiomics to predict survival in high-grade serous ovarian cancer.

Two papers incorporate a measure of mutation burden of the genome in their predictive models. Veeraraghavan et al.^34^ use copy number burden as a measure of genomic instability and combine it with clinical and radiomics features to predict progression-free survival and platinum resistance in high grade serous ovarian carcinoma. Vanguri et al.^39^ study both specific genes with known predictive power (such as EGFR) as well as the tumour mutation burden (TMB) to predict response to immunotherapy in non-small cell lung cancer. They show that the performance of a logistic regression model which uses genomics without TMB is inferior to the model trained using genomics with TMB. They also combine TMB and genomics data with radiomics and computational pathology to develop a combined deep learning model.

## Data translation

**Fig. 3:**
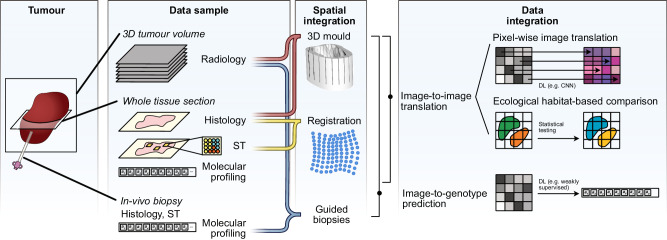
Schematic of data translation process. From left to right: types of data samples extracted from tumours, spatial integration techniques for sample co-registration, and data integration techniques for cross-modal translation, including image-to-image translation and image-to-genotype prediction. ST spatial transcriptomics.

Data fusion leverages the complementarity between data types to produce better overall predictions. However, its focus is not on dissecting the complex interactions between the data types, which could bring additional understanding about the disease. Data translation bridges the gap between different types of data, allowing for insights that might not be apparent when viewing each data type in isolation (Fig. 3).

In particular, data translation underpins the majority of approaches aiming to assign biological explanation to radiomics features. This explainability has been argued to be crucial for clinical application and acceptance of radiomics, with several classes of data suggested as biological correlates (e.g. genomic data, IHC, pathological images and habitat imaging)^43^.

### Predicting genotype from image-omics

The tumour phenotype, as it is expressed histologically and radiologically, is an indirect reflection of the underlying molecular landscape. In many cases, the genotype directly affects the tumour phenotype giving rise to distinct morphological patterns. This is particularly apparent in histopathology and has led to the development of many computational pathology deep learning algorithms which directly predict key mutations from routinely acquired tissue samples. These algorithms extract morphological features from H&E slides and have shown good accuracy in predicting various types of mutations. Kather et al.^44^ use a weakly-supervised method with attention mechanism to predict microsatellite instability in gastrointestinal cancer patients from H&E images with good accuracy. The attention mechanism of the approach allows for the mapping of the network’s attention scores showing the areas which are considered important by the model. Similar concepts are also exploited by Fremond et al.^45^ where an attention-based deep learning algorithm sorts endometrial cancer patients into four molecular subgroups, and by Lu et al.^46^, where a graphical network predicts HER2 mutation in breast cancer patients with high accuracy. Technologies such as spatial transcriptomics can provide full molecular expression analysis at the resolution of few-cells while retaining their spatial context. Recent studies showcase the feasibility of deep learning to predict the spatial transcriptomics profile from H&E samples with high accuracy^47–49^.

In the area commonly known as ‘radiogenomics’, models trained from quantitative features (radiomics) extracted from standard-of-care radiological images are used to predict the underlying genotype, thus offering a non-invasive method to gain insights into the tumour’s molecular profile. Researchers have shown promising results of radiomics-based models predicting driver mutations in many cancer types, including lung^29^, breast^50^, colorectal^51^ and clear cell renal cell carcinoma^52^. However, the routine clinical adoption of radiomics approaches remains difficult mainly due to suboptimal robustness in multi-centric unseen data and lack of understanding of the tumour biology that guides the model’s prediction. A link between imaging and the underlying histology could be the key in providing confidence and the necessary explainability to the genotypic predictions of radiogenomics-based models.

### Predicting histology from imaging

In the UK imaging biomarker roadmap, O’Connor et al.^53^ state that imaging biomarkers require stringent validation before they can be clinically deployed. However, histological validation is not a trivial task. Registering the three-dimensional (3D) MRI volume to the two-dimensional histology slides is difficult, due to low out-of-plane resolutions, histology tissue deformations, unknown orientations, and lack of details or landmarks in the radiology images. An elegant solution is to create 3D patient-specific moulds and use a microtome to cut the tumours and acquire slides co-aligned to the radiology slices. 3D moulds have been successfully applied in various cancer types, such as prostate^54,55^, ovarian^56^ and renal tumours^57^ and breast cancer mouse models^58^. After imaging-histology co-alignment is achieved, registration is required to increase the accuracy of the matching between the two modalities.

At that stage, computational pathology can be used to spatially correlate medical imaging information with histopathological features, thereby providing explainability and confidence to the imaging measurements. Jardim-Perassi et al. use 3D printed tumour moulds to obtain co-registered MRI and histology in breast cancer mouse models. Subsequently, they use a commercial digital pathology software platform to process H&E and IHC samples and spatially histologically validate four tumour habitats identified directly from multi-parametric MRI: viable-normoxic, viable-hypoxic, nonviable-normoxic and nonviable-hypoxic^58^. Zormpas-Petridis et al.^59^ use an automatic AI cell-classification algorithm on co-registered H&E samples to associate high T1 mapping MRI values with high densities of aggressive undifferentiated neuroblasts in a transgenic mouse model of high-risk neuroblastoma.

Despite these promising results, tumour mould development is a complex process, with more research needed before it can be included in standard clinical practice. On a technical level, ensuring the precision of radiological image segmentation, tumour positioning in the mould and histology-to-radiology slice co-registration is challenging, particularly in tumours without clear anatomical landmarks. Some of the proposed solutions include annotating the mould base position at the segmentation stage and cutting an additional ‘orientation incision’ at a pre-defined location relative to the lesion base^56^, or, in the case of highly deformable organs such as the liver, employing a mould that completely surrounds the sample, which can help prevent changes in shape during slicing^60^. More recently, alternative approaches have been proposed that eliminate the need for moulds by letting a deep learning based co-registration model find the optimal alignment between tissue sections and 3D scan slices^61^.

### Virtual biopsies

Despite the crucial information about the histological, molecular and genetic properties of the tumour that is obtained from surgical biopsies, a biopsy sample represents only a “snapshot” of the tumour at a given time or location. Monitoring tumour evolution would require multiple repeated biopsies, which is often not feasible and is limited by multiple complications. Additionally, the highly heterogeneous nature of many tumour types offers no guarantees that the biopsy sample is representative of the entire tumour. This was clearly demonstrated in the multi-region TRACERx cohort, where the existence of “immune-cold” regions identified by computational pathology was independently prognostic for survival despite other regions of the tumour being “immune-hot”^62^.

With advancements in imaging techniques and computational methodologies, the concept of “virtual biopsies” has gained traction^63^. By interconnecting imaging data at a spatial level with both molecular and histological information, it is possible to infer the internal characteristics of a tumour without physically extracting tissue samples. This approach is not only less invasive but can also provide longitudinal real-time insights into tumour heterogeneity and evolution. Although the length scales of radiology do not allow the imaging of single cells, they can characterise the regions (“habitats”) in which they reside, potentially revealing the spatial variations in the density of different cell population^59,64^ and inform on the underlying conditions, such as tumour metabolism and blood flow^65,66^. Virtual biopsies can also be used to guide radiotherapy treatment to target the most aggressive tumour areas and inform on more meaningful sites for surgical biopsies^67^. Identifying and monitoring tumour habitats can also lead to the early detection of emergence to treatment resistance^68^.

The successful deployment of virtual biopsies requires a spatial streamline link between the radiological images, the histological samples and genomic/transcriptomic data. Beer et al.^67^ incorporate a CT/ultrasound fusion to guide biopsy sampling from radiomics-defined tumour habitats. Additionally, Weigelt et al.^69^ demonstrate in a patient with high-grade ovarian cancer that radiomics-defined image habitats present with distinct histological and molecular patterns.

## Data aggregation

**Fig. 4:**
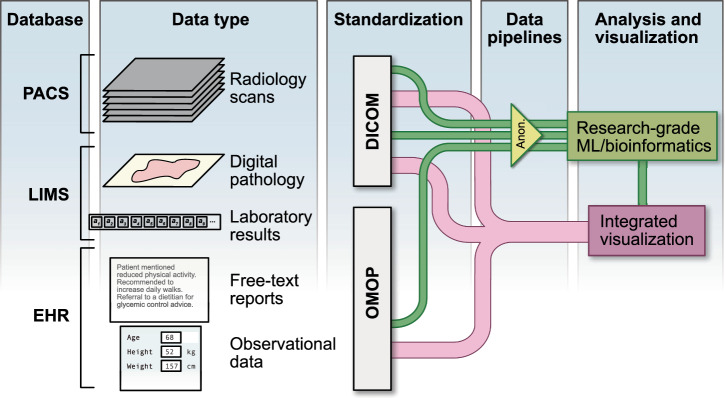
Data aggregation. Schematic of the data aggregation process to connect clinical information from Electronic Health Records (EHRs), imaging modalities from Picture Archiving and Communication Systems (PACS), and molecular markers from Laboratory Information Management Systems (LIMS), including a standardisation step for interoperability. Data can be processed offline after anonymisation (green pipelines) or displayed and visualised in real time (pink pipelines). Models trained on anonymised data can be deployed and integrated as part of live visualisation tools.

The third and final type of data integration, namely data aggregation, refers to the systematic process of collecting, preparing, and presenting diverse datasets for analysis (Fig. 4). Even though it is a step that is often left out of the data integration discussion, without it clinical models that rely on data integration, particularly if they contain images, could not be deployed. The objective is to unify various data streams, including clinical information from Electronic Health Records (EHRs), imaging modalities from Picture Archiving and Communication Systems (PACS), or molecular biomarkers from Laboratory Information Management Systems (LIMS).

### Data frameworks

One of the foundational steps in data aggregation involves establishing structured frameworks that standardise data formats, ensuring uniformity and simplifying subsequent integration processes.

Of particular interest for healthcare-focused data integration is the adoption of data models, acting as a universal translator to facilitate cross-organisation data exchange and utility. For example, the Observational Medical Outcomes Partnership (OMOP) Common Data Model^70^ seeks to standardise the structure and content of observational data to facilitate robust and reproducible downstream analyses, no matter which organisation was responsible for generating the data. For imaging, the DICOM format is widely used for radiological imaging^71^, and is also gaining traction in digital pathology^72^. One of the strengths of DICOM format is the incorporation of metadata in the files, ensuring that this additional layer of information is directly linked to the pixel data. Metadata can be critical for accurate analysis and comparison across different datasets, for example to ensure that there is no systematic bias arising from differences in equipment settings or choice of machine manufacturer^73^. More research is being conducted on automated metadata interpretation, in order to more easily identify desired image characteristics, e.g. MRI sequences^74^.

### Offline analysis

Aggregation and transformation of multimodal data into coherent and linked sets of data ready for further use requires robust data processing pipelines. In the research setting, a common challenge is the de-identification of datasets and production of ‘minimum viable datasets’ to ensure that participant anonymity is preserved. Some modalities, like free text, can be very challenging to de-identify, with results that are often inaccurate and unreliable^75^. Deep learning based natural language processing methods have also been proposed in recent years for de-identification purposes^76^ and beyond. They are of particular interest in imaging given the duality of the data—images coupled with free text reports—and have showed promise in various tasks which could facilitate data aggregation. For example, large language models (LLMs) have been successful in transforming free text radiology reports into impression statements^77^ and structured reports^78^, both of which are more efficient data formats for further analysis. Furthermore, LLMs have also been used to extract labels from radiology reports, such as labels of oncological progression or diagnosis of metastatic disease^79^, which shows their potential in automating the step of patient classification in data aggregation workflows. This step will likely also benefit from developments in a related method: vision-language processing, where the model includes both a text encoder and an image encoder; this allows for self-supervised learning from image-report pairs, with new research incorporating a temporal aspect to the process^80^.

There are a number of unique challenges to de-identification of radiology data. Within the DICOM format, metadata need to be anonymised without affecting the diagnostic relevance of the images, which might be ambiguous, for example when dealing with scan dates, manufacturers’ private tags or institution names. The reliability of de-identification tools has also been questioned in literature—in 2015, Aryanto et al.^81^ reviewed 10 free toolkits and found that full anonymisation was achieved by only one of them with default settings, and four with manual adjustment of settings. However, a more recent review of available techniques is needed. A further challenge of radiological image anonymisation is posed by the process of embedding identifiable data into the pixel image as “burned-in” text, which might be difficult to detect and anonymise without removing the full image from the file; new techniques based on optical character recognition are being developed to tackle this issue^82^.

### Live deployment

Data integration models designed to be deployed in clinical practice rely on all the data streams being available simultaneously and in a timely fashion. However, historically EHR and PACS systems were developed independently, and most radiology departments do not have PACS-EHR integration^83^ even though existing data suggest that it significantly improves productivity, even in the absence of novel AI-based support tools^84^.

## Discussion and outlook

The inclusion of medical imaging in multi-omic models for precision oncology brings challenges and opportunities.

Our review of the literature in the imaging-omics data fusion space showed that the field is still young and has not yet exploited the latest developments in AI research. Most studies integrate few data sources and use a late fusion approach in which most of the analysis is done for each data type independently. Many studies still rely on handcrafted features and the manual selection of interesting biomarkers. This is not as suboptimal as it may sound; a few well selected features guided by a domain expert could summarise information that would otherwise be very expensive for an unbiased deep learning framework to learn. Similarly, given the practical challenges of data aggregation for live deployment of data fusion models in the clinical setting, simplified models may be generally preferable. Nevertheless, our literature search has limitations. We limited ourselves to papers that studied response, prognosis, or outcome, and excluded other areas of data fusion such as diagnosis. Even though we did not actively exclude them, we did not specifically search for integration with clinical reports and medical records either—a field that is now receiving considerable attention with the recent success of large language models. In addition, we looked for specific terms to identify the different data modalities integrated in the study; as a result, we may have missed papers with a broader remit, such as those more interested in the method development than the application.

A unique feature of the integration of medical images is the introduction of a new physical dimension that provides context to the other data modalities. Data translation studies are making significant progress in this area, with the ultimate vision of generating a true multi-scale atlas, spanning from macroscopic radiology features down to the cellular microenvironment. Data fusion could also benefit from similar ideas of spatial co-localisation. For example, intra- and inter-tumour heterogeneity is a well known feature of many cancers, and yet data fusion models do not encode spatial alignment between biopsies and radiological location, which could lead to significant confounding effects.

Finally, deploying any of these models in a clinical scenario will generally require significant technological investment, as clinical systems are not necessarily designed for effective data aggregation. As the popularity of AI-assisted digital tools grows, this problem is likely to get progressively resolved.

### Supplementary information

Supplementary information
