# Supplementary material for: Radiology and multi-scale data integration for precision oncology
Source: NPJ Precis Oncol. 2024 Jul 26;8:158. doi: 10.1038/s41698-024-00656-0 (PMC11282284; doi:10.1038/s41698-024-00656-0)
Supplement: Supplementary file 1 — Supplementary information [file 41698_2024_656_MOESM1_ESM.pdf]

Supplementary Information

A PubMed search

Our literature search was performed on MEDLINE (PubMed) with query terms showed in Supplementary Table 1. The search was conducted on 18th June 2023.

Supplementary Table 1: PubMed search strategy.

Search number	Query	Result
8	(#7) NOT (review[Publication Type])	27
7	#6 AND "cancer"[tiab]	46
6	#5 AND ("response"[tiab] OR "outcome"[tiab] or "prognos*"[tiab])	92
5	#1 AND (#2 OR #3) AND #4 AND integ*	201
4	("Machine Learning"[Mesh]) OR ("machine learning"[tiab]) OR "deep learning"[tiab] OR "AI"[tiab] OR "artificial intelligence"[tiab]	195,222
3	("Genomics" [Mesh] OR genomic* [tiab] OR "Mutation" [Mesh] OR mutation* [tiab] OR "clinicogenomic*"[tiab] OR "clinico-genomic*"[tiab] OR "clinical-genomic*"[tiab])	1,576,476
2	(((transcriptomic*[tiab]) OR (metabolomic*[tiab])) OR ("molecular biomarker*"[tiab])) OR (proteomic*[tiab]) OR "digital pathology"[tiab] OR "pathomic*"[tiab] OR "computational pathology"[tiab]	207,038
1	("texture analys*" [tiab] OR radiomic*[tiab] OR radiolog*[tiab] OR "CT scan*"[tiab] OR "CT imag*"[tiab] OR "computed tomography"[tiab] OR MRI[tiab] OR "MR imag*"[tiab] OR "MR scan*"[tiab] OR "magnetic resonance*"[tiab])	1,204,374

B Search results

Supplementary Table 2: Summary of all the studies using data fusion and medical imaging to predict treatment response or long-term outcome identified in the PubMed search.

Reference	Task	Tumour type	Number of data types	Data types	Integration model
Wang et al., 2022 [?]	Survival prediction	Colorectal cancer	4	H&E, Radiomics, IHC, Clinical	Cox regression nomogram
Feng et al., 2022 [?]	Treatment response prediction	Rectal cancer	2	Radiomics, H&E	Support Vector Machine
Chabon et al., 2020 [?]	Excluded - Early detection	Non small cell lung cancer	1	Genomics	N/A
Boehm et al., 2022 [?]	Survival prediction	High-grade serous ovarian cancer	4	Radiomics, H&E, Genomics, Clinical	Multivariate Cox model
Wang et al., 2022 [?]	Treatment response prediction	Lung cancer	2	Radiomics, Clinical	Support Vector Machine
Vanguri et al., 2022 [?]	Treatment response prediction	Non-small cell lung cancer	2	Radiomics, Pathology, Genomics	Deep learning
Migliozzi et al., 2023 [?]	Excluded - Multi-omic tumour subtype characterisation	Glioblastoma	9	Transcriptomics, Proteomics, Metabolomics, Lipidomics, Acetylomics, Genomics, Radiomics, Clinical	Indirect approach
Safari et al., 2022 [?]	Excluded - Genetic mutation status prediction	Glioma	1	Radiomics	N/A
Zheng et al., [?]	Excluded - Genetic mutation status prediction	Lung cancer	1	Radiomics	N/A
Chen et al., 2021 [?]	Excluded - Genetic mutation status prediction	Glioma	1	Radiomics	N/A
Bensoussan et al., 2023 [?]	Excluded - Review paper	N/A	N/A	N/A	N/A
Cardoso et al., 2022 [?]	Treatment response prediction	Breast cancer	2	Radiomics, Clinical	Logistic regression
Zhang et al., 2021 [?]	Prediction of microsatellite instability status	Rectal cancer	2	Radiomics, Clinical	Deep learning
Zhou et al., 2019 [?]	Prediction of genetic mutation status	Glioma	2	Radiomics, Clinical	Random forest
Pachynski et al., 2021 [?]	Excluded- Multi-omic tumour subtype characterisation	Prostate cancer	2	IHC, Transcriptomics	Indirect approach
Pei et al., 2021 [?]	Excluded - Tumour grade prediction	Glioma	2	H&E, Genomics	Deep learning
Iwatate et al., 2020 [?]	Genetic mutation status prediction Indirect survival prognosis prediction	Pancreatic ductal adenocarcinoma	2	Radiomics, Clinical	Indirect approach
Veeraraghavan et al., 2020 [?]	Survival and treatment response prediction	High grade serous ovarian carcinoma	2	Radiomics, Clinical, Genomics	Support Vector Machine
Zeng et al., 2021 [?]	Survival prediction	Clear cell renal cell carcinoma	4	Radiomics, Genomics, Transcriptomics, Proteomics	Random forest
Hoivik et al., 2021 [?]	Survival prediction	Endometrial cancer	3	Radiomics, Clinical, Transcriptomics, Genomics	Indirect approach
Yi et al., 2021 [?]	Treatment response prediction	Ovarian cancer	3	Radiomics, Genomics, Clinical	LASSO, random forest, SVM
Cook et al., 2023 [?]	Treatment response prediction	Breast cancer	2	Radiomics, Transcriptomics	Computational biophysics simulation model
Li et al., 2023 [?]	Survival prediction	Gastric cancer, glioblastoma & low grade glioma	2	Radiomics, H&E	Deep learning
Gallivanone et al., 2019 [?]	Excluded - Tumour pathological type prediction	Breast cancer	2	Radiomics, Transcriptomics	Support Vector Machine
Bateman et al., 2016 [?]	Excluded - abstract book	N/A	N/A	N/A	N/A
Cavalieri et al., 2021 [?]	Excluded - report of database creation, not a research study with machine learning model	N/A	N/A	N/A	N/A
Liang et al., 2023 [?]	Survival prediction	Clear cell renal cell carcinoma	3	Radiomics	Indirect approach
